# Plasma metabolomic characterization of SARS-CoV-2 Omicron infection

**DOI:** 10.1038/s41419-023-05791-3

**Published:** 2023-04-19

**Authors:** Xue Li, Yimeng Liu, Guiying Xu, Yi Xie, Ximo Wang, Junping Wu, Huaiyong Chen

**Affiliations:** 1 Department of Basic Medicine, Haihe Clinical School, Tianjin Medical University, Tianjin, 300350 China; 2 Department of Basic Medicine, Haihe Hospital, Tianjin University, Tianjin, 300350 China; 3 Tianjin Key Laboratory of Lung Regenerative Medicine, Tianjin, 300350 China; 4 Key Research Laboratory for Infectious Disease Prevention for State Administration of Traditional Chinese Medicine, Tianjin Institute of Respiratory Diseases, Tianjin, 300350 China; 5 Tianjin Key Laboratory of Acute Abdomen Disease Associated Organ injury and ITCWM Repair, Tianjin, China; 6 Department of Tuberculosis, Haihe Hospital, Tianjin University, Tianjin, 300350 China

**Keywords:** Viral infection, Inflammatory diseases

## Abstract

Omicron variants of SARS-CoV-2 have spread rapidly worldwide; however, most infected patients have mild or no symptoms. This study aimed to understand the host response to Omicron infections by performing metabolomic profiling of plasma. We observed that Omicron infections triggered an inflammatory response and innate immune, and adaptive immunity was suppressed, including reduced T-cell response and immunoglobulin antibody production. Similar to the original SARS-CoV-2 strain circulating in 2019, the host developed an anti-inflammatory response and accelerated energy metabolism in response to Omicron infection. However, differential regulation of macrophage polarization and reduced neutrophil function has been observed in Omicron infections. Interferon-induced antiviral immunity was not as strong in Omicron infections as in the original SARS-CoV-2 infections. The host response to Omicron infections increased antioxidant capacity and liver detoxification more than in the original strain. Hence, these findings suggest that Omicron infections cause weaker inflammatory alterations and immune responses than the original SARS-CoV-2 strain.

## Introduction

Infections with early strains of the severe acute respiratory syndrome coronavirus 2 (SARS-CoV-2) led to mild-to-severe disease in 2020. Based on WHO Coronavirus (COVID-19) Dashboard, global cases of patients with coronavirus disease 2019 (COVID-19) have increased by 370 million by November 28, 2022, since the identification of SARS-CoV-2 Omicron strain in November 2021 [1]. In the last year, the number of SARS-CoV-2 Omicron cases has exceeded 30% of all patients with the initial SARS-CoV-2 strain that emerged in 2019 and variants in 2020 and 2021. However, the mortality rate of SARS-CoV-2 Omicron cases has dropped significantly, which is consistent with the general laws of viral evolution and phylogeny during human transmission. Most patients with Omicron developed no or mild symptoms and did not require ICU care regardless of their vaccination status [2]. Compared to the original strain and previous variants, clinical manifestations of Omicron infections were mostly restricted to the upper airways; however, they showed more symptoms, including cough, expectoration, nasal congestion, runny nose, and hoarse voice [3, 4]. Omicron has more mutations than previous strains, and mutations within the receptor-binding domain of the spike protein stabilize the conformation of the spike and therefore restrict the accessibility of neutralizing antibodies [5]. Reduced neutralization from previous vaccinations contributes to its rapid spread and viral shedding in asymptomatic cases [6, 7]. Clinical and hematological parameters clearly demonstrated that Omicron reduced pathogenicity [8]. However, it remains unclear whether reduced pathogenicity of Omicron infections is reflected in the host response compared to previous variants.

Metabolomics has provided a powerful platform for revealing the molecular mechanisms underlying the pathogenesis of infections with early SARS-CoV-2 strains. The downregulation of glycerophospholipids, sphingolipids, and fatty acids in the sera of patients with non-severe or severe COVID-19 revealed liver injury [9]. In these patients, elevating glucose glucoronate and a bilirubin degradation product suggest a potentially impaired liver detoxification function. Su et al. [10] discovered a clear downregulation trend in amino acid and lipid metabolism in patients with severe COVID-19 infection; however, not in mild or moderate cases. In this study, a metabolic shift was identified in patients with mild to moderate disease status with inflammation. Dysregulated circulating metabolites associated with glucose metabolism and the urine cycle may be related to susceptibility, severity, and recovery in patients with SARS-CoV-2 infection [11]. Several circulating lipids, including phosphatidylcholine, phosphatidylethanolamine, arachidonic acid, and oleic acid, act as potential biomarkers for infection (for example SARS-CoV-2, Zika virus, and Schistosoma haematobium) and disease severity [12–14]. These findings suggest that serum metabolomics may provide insights into the host response to infection by various SARS-CoV-2 strains.

We previously revealed metabolic alterations in patients with SARS who survived 12 years after discharge and those with SARS-CoV-2 who survived 6 months after discharge [15, 16]. In this study, we used a quantitative metabolomic approach to analyze plasma samples obtained from healthy individuals and patients infected with the Omicron variants with non-severe symptoms. Moreover, we compared the metabolomic features of Omicron infection with non-severe symptoms with those of four existing reports on non-severe COVID-19 in patients infected with the original SARS-CoV-2 strain circulating from 2019. Our data suggest decreased adaptive immunity and increased anti-inflammatory efficiency in patients with non-severe COVID-19 with Omicron infections than in healthy individuals. Compared with the original SARS-CoV-2 strain, Omicron infections in patients induced weaker neutrophil functions and interferon-induced immunity. However, it also induced a stronger anti-oxidative response and liver detoxification.

## Results

### Demographic and clinical features of patients with COVID-19

Between January and March 2022, 417 patients with Omicron infection were admitted to Haihe Hospital and First Central Hospital (Tianjin, China), from which we successfully obtained plasma from 19 patients with non-severe symptoms during the acute infection period and 31 patients with non-severe symptoms who had recovered. We collected clinical information from these patients with COVID-19 and 33 healthy control participants. We observed that the mean age of infected patients was 40.6 ± 14.2 years; 7 (36.8%) were men, and 12 (63.2%) were women. Further, the mean age of recovered patients was 45.6 ± 14.7 years; 16 patients (51.6%) were men, while 15 (48.4%) were women. All the patients presented with mild and moderate COVID-19 cases, 10 patients (52.6%) were mild, 9 patients (47.4%) were moderate in the infected group, 8 patients (25.8%) were mild, and 23 patients (74.2%) were moderate in the recovered group. The inpatient days were 13.8 ± 4.7 days for the infected group and 12.7 ± 4.9 days for the recovered group. We also matched 33 healthy participants with similar demographic characteristics to establish a healthy control group. The demographic and clinical information of all the participants is summarized in Table 1.

**Table 1:** Demographics and clinical characteristics of COVID-19 patients.

Characteristics	Healthy control (*n* = 33)	COVID-19 (*n* = 52)	
		Infected (*n* = 19)	Recovered (*n* = 31)
Sex–no. (%)			
Male	18(54.5)	7 (36.8)	16 (51.6)
Female	15(45.5)	12 (63.2)	15 (48.4)
Age–year			
Mean ± SD.	41.2 ± 8.8	40.6 ± 14.2	45.6 ± 14.7
Range	25.0–64.0	16.0–67.0	19.0–69.0
Severity–no. (%)			
Mild		10 (52.6)	8 (25.8)
Moderate		9 (47.4)	23 (74.2)
Severe		0 (0.0)	0 (0.0)
Critical		0 (0.0)	0 (0.0)
Inpatient days			
Mean ± SD.		13.8 ± 4.7	12.7 ± 4.9
Range		7.0–29.0	6.0–22.0

### Plasma metabolomic profiling of COVID-19 patients

**Fig. 1:**
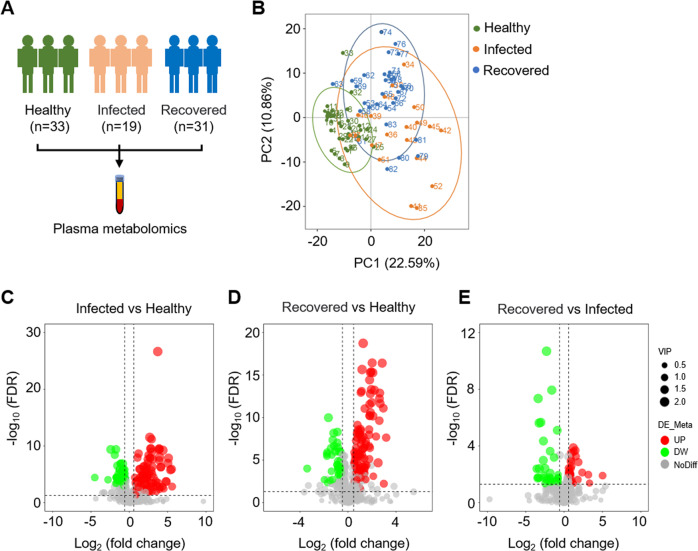
Overview of the study design. **A** Schematic summary of study design and participants. **B** PCA diagram of metabolomic data from plasma samples. Each dot represents a plasma sample, and different colors mark different groups. The green, orange, and blue data points were the healthy control group, the infected group, and the recovered group, respectively. **C**–**E** Displays volcano plots comparing three pairs of groups. Each point in the volcano plots represents a metabolite. The metabolites with FDR < 0.05 were considered to be significant differences. Red represents the upregulated metabolites; green represents the downregulated metabolites. The size of the dot represents the VIP value.

We used a non-targeted metabolomics approach to analyze plasma from 83 individuals (Fig. 1A). Overall, 546 metabolites were identified based on compound libraries (mzCloud, mzVault, and MassList). PCA of metabolites revealed a clear distinction between patients with COVID-19 (including infected and recovered groups) and healthy controls. The difference between the infected and recovered groups was minimal (Fig. 1B). One hundred sixty-seven differentially expressed metabolites (DEMs) were identified between the infected and healthy control groups, with 105 upregulated and 62 downregulated (Fig. 1C). In recovered patients compared with healthy controls, 150 significant DEMs were detected, of which 112 were upregulated, and 38 were downregulated (Fig. 1D). There were 78 DEMs between the recovered and infected groups, with 44 upregulated and 34 downregulated DEMs (Fig. 1E).

**Fig. 2:**
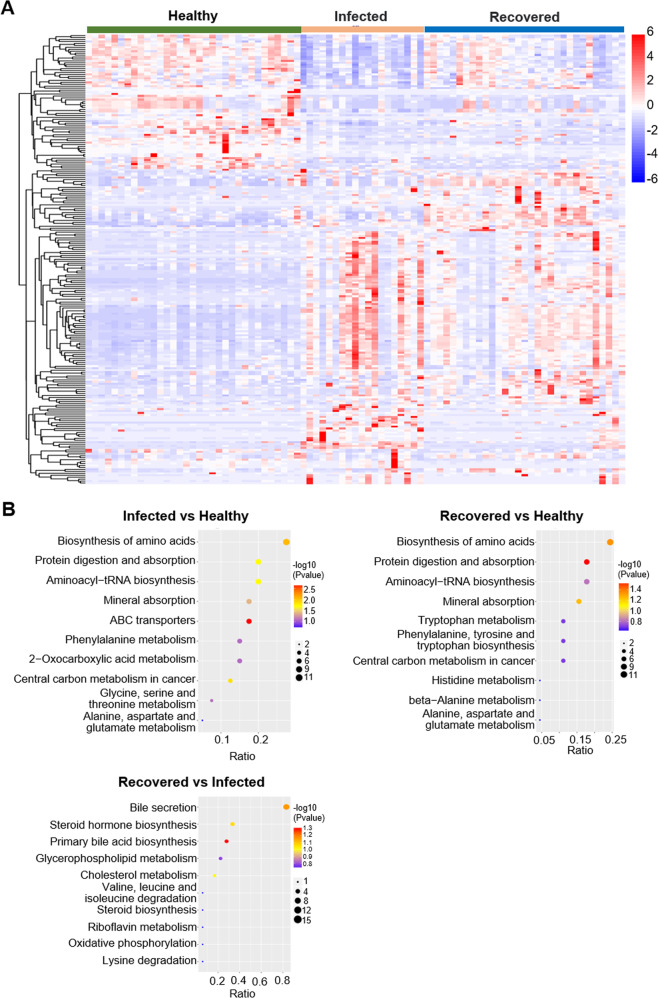
Metabolomic profiling of plasma samples obtained from patients with Omicron and healthy participants. **A** Heatmap visualization of significantly different altered metabolites (DEMs) in infected, recovered patients and healthy control participants. Metabolites in the heatmap showed a fold-change >1.5 and FDR < 0.05. The color bar represents the relative intensity of identified metabolites from −6 to 6. **B** Bubble plots show the top 10 KEGG enrichment pathways. The ratio is the number of differential metabolites in the related metabolic pathway to the number of total metabolites identified in the pathway. The color of the dots represents −log_10_ (*p* value). The size of the dots represents the number of differential metabolites in the corresponding pathway.

The DEM visualization heatmap shows altered metabolites (Fig. 2A, Table S1–S3). In addition, KEGG enrichment analysis revealed that the DEMs played major roles in the biosynthesis and metabolism of amino acids and protein digestion and absorption in patients with COVID-19 (including infected and recovered groups) and healthy controls (Fig. 2B, Table S4–S5). However, compared to infected patients, DEMs played major roles in bile acid biosynthesis and secretion, glycerophospholipid metabolism, cholesterol metabolism, and other processes in recovered patients (Fig. 2B, Table S6).

### Altered amino acids metabolism and disordered immune responses

**Fig. 3:**
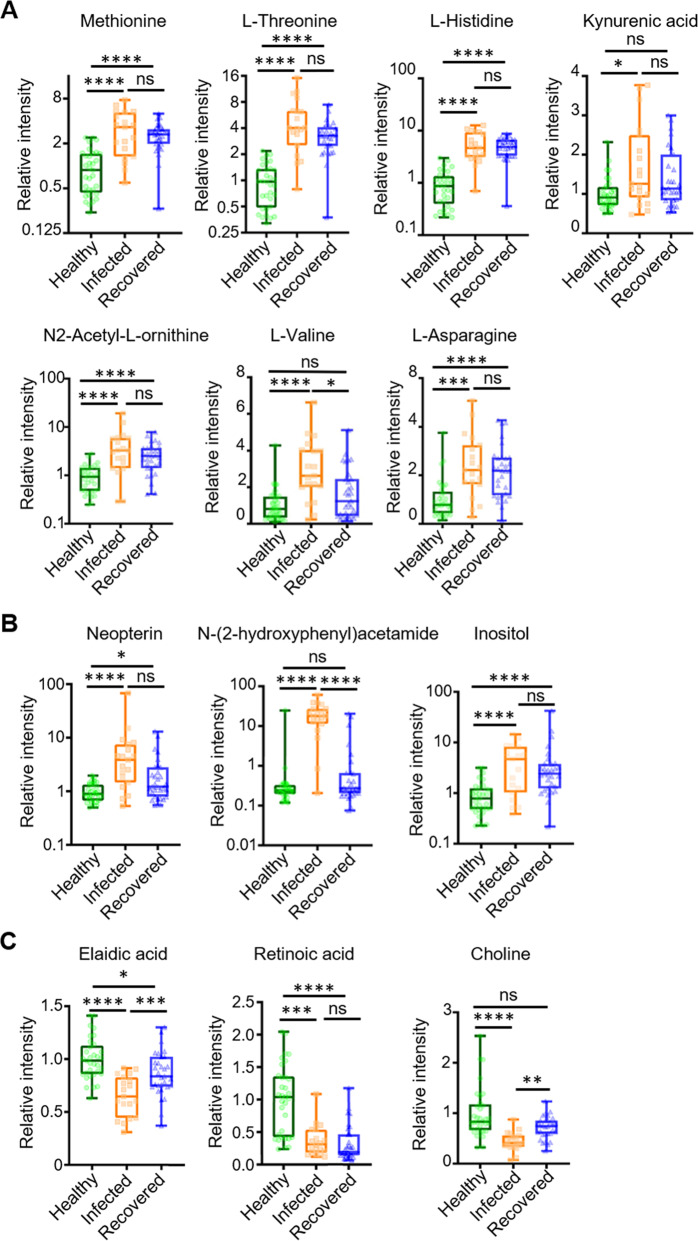
Differential metabolites between patients with Omicron patients and healthy participants. For the healthy control group, *n* = 33; for the infected group, *n* = 19; and for the recovered group, *n* = 31. ns: nonsignificant. *p < 0.05, **p < 0.01, ***p < 0.001, ****p < 0.0001.

Various amino acid metabolites, including methionine, L-threonine, L-histidine, L-asparagine, and N2-acetyl-L-ornithine, were found to be upregulated in patients with COVID-19, including the infected and recovered groups (Fig. 3A). L-valine and kynurenic acid returned to normal levels in recovered patients. In contrast, abnormalities in amino acid levels reflect increased protein catabolism in patients; amino acids are also involved in immune regulation. Methionine metabolism was induced in activated T cells and methionine restriction reduced T-cell proliferation and cytokine secretion via epigenetic regulation [17]. In our results, plasma methionine accumulation may reflect a decrease in the usage of this amino acid for T-cell activation (Fig. 3A). Histidine elevation indicated that the inflammatory response to Omicron infection was inhibited because histidine has been shown to block oxidative stress and downregulate TNF-α in neutrophils [18, 19]. T-cell-mediated immunity seemed downregulated because kynurenic acid positively correlated with inflammatory cytokines and chemokines and negatively correlated with T-cell responses [20, 21]. Notably, N2-acetyl-L-ornithine and asparagine, which are ornithine cycle-related metabolites, are significantly upregulated in patients with severe COVID-19 and highly correlated with the “cytokine storm“ [11, 22, 23]. In contrast to previous studies [12, 24], the infected group had higher levels of threonine and L-valine. L-valine is also involved in the biosynthesis of pantothenate and coenzyme A, which are essential for mitochondrial energy [25].

In addition, patients with COVID-19 had increased levels of metabolites associated with the immune response of the body, including neopterin, N-(2-hydroxyphenyl) acetamide, and inositol were observed in patients with COVID-19 (Fig. 3B). Neopterin production is directly related to immune cell activation. Neopterin is an antioxidant produced by IFN-γ-stimulated mononuclear phagocytes [26]. Neopterin levels are elevated in various diseases, such as cancer, autoimmune diseases, and viral infections, including COVID-19 [27, 28]. Neopterin levels were significantly increased in patients with COVID-19 [27, 29, 30]. Furthermore, they showed a weak negative correlation with the number of days after onset in patients with COVID-19 [30], suggesting that a neopterin elevation is an early event of viral infection. N-(2-hydroxyphenyl) acetamide has anti-inflammatory properties by reducing IL-1β and TNF-α inflammatory factors expressions [31, 32]. Inositol is a phospholipid surfactant precursor and is used to stimulate surfactant synthesis. It downregulates the IL-6 expression through the phosphatidylinositol-3-kinase pathway and reduces the inflammatory cascade [33–35].

Infected patients had significantly lower levels of choline, elaidic acid, and retinoic acid than the healthy controls (Fig. 3C). Metabolomic studies of patients with COVID-19 have also reported abnormal choline levels and its derivatives [9, 12]. This was probably because of activated macrophage-mediated immunity [36]. The polarization of macrophages in response to pathogens necessitates increased absorption of choline, which promotes cytokine secretion [36]. Elaidic acid levels were lower in extracellular vesicles isolated from the serum of patients with COVID-19 [37]. In addition, elaidic acid stimulates the release of inflammatory factors in macrophages [38]. Retinoic acid is a vitamin A metabolite that stimulates the production of antibodies by B cells [39, 40]. Previous studies have revealed that all-trans retinoic acid (ATRA), a retinol derivative, exhibits antiviral activity against several viruses by enhancing IFN-I-mediated viral clearance [41]. ATRA showed potent antiviral activity against SARS-CoV-2 infection in human cell lines and organoids [42]. These findings suggest that in response to Omicron infections, the host develops antiviral inflammation, not adaptive immunity.

### Distinct profiles of infected and recovered patients

**Fig. 4:**
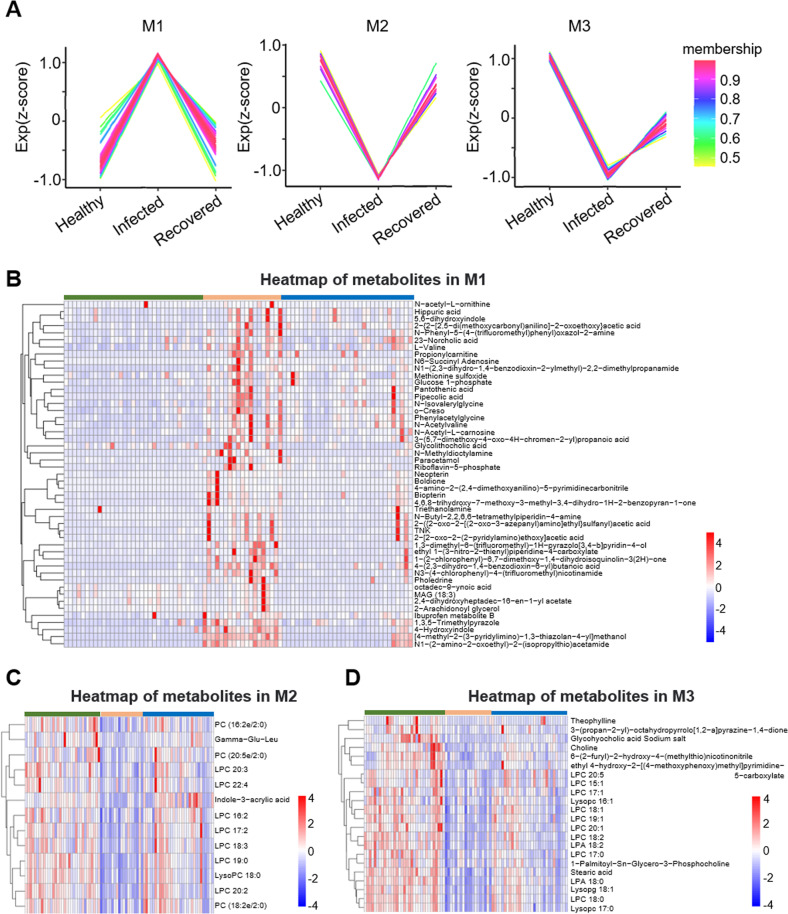
Expression profiles were analyzed according to metabolic abundance between patients with COVID-19 and healthy participants. **A** The result of cluster analysis in processing conditions (Healthy-Infected-Recovered) by the Mfuzz package, M1–M3. The color bar represents the *Z* score change from −1 to 1. **B**–**D** Heatmap visualization of metabolites in M1–M3 under the processing conditions (healthy, infected, recovered). The color bar represents the relative intensity of identified metabolites from −4 to 4.

The Mfuzz package was used to process the metabolic abundance matrix, revealing the presence of six clusters (Fig. 4A, Fig S1A). We observed that three sets of metabolites showed that recovered patients tended to attain the status of healthy control (49 metabolites in M1 set, 13 metabolites in M2 set, and 22 metabolites in M3 set) (Fig. 4B–D). There were 142 metabolites that did not return to normal levels (64 metabolites in M4 set, 45 metabolites in M5 set, and 33 metabolites in M6 set) (Fig S1C). In addition, we discovered various organic acids, derivatives, lipids, and lipid-like molecules in the M1 metabolite set, which was significantly upregulated in the infected group. KEGG enrichment analysis revealed that these metabolites played major roles in pantothenate and CoA biosynthesis, vitamin digestion and absorption, amino acid metabolism, and other processes (Fig. S1B). Moreover, we found that the majority of metabolites expressed in the M2 and M3 sets were mainly glycerophospholipids (11 compounds in M2 and 14 compounds in M3), which were decreased in the infected group; however, they significantly increased in the recovered group. KEGG enrichment analysis revealed that these metabolites played major roles in choline metabolism, glycerophospholipid metabolism, fatty acid biosynthesis, and other processes (Fig. S1B).

### Differential host response to Omicron versus the original SARS-CoV-2 strain

**Fig. 5:**
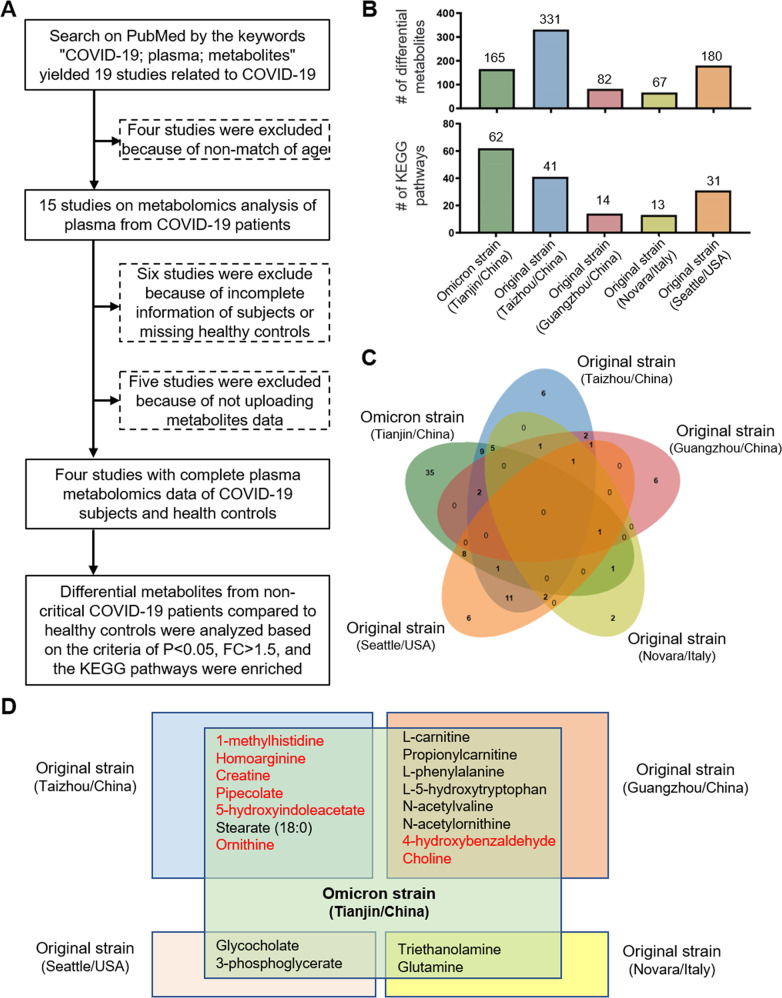
Comparison of plasma metabolomics of Omicron infection with the original strain. **A** Flowchart for screening published articles of plasma metabolomic analysis in patients with COVID-19. **B** The number of differential metabolites and KEGG enrichment pathway in patients with COVID-19 infected with Omicron and the original strains. **C** The Venn diagram compares enrichment pathways between the Omicron and the original strains. **D** Shared differential metabolites in patients infected with the Omicron and the original strains, respectively. The red metabolites present the opposite change from Omicron patients to the original strain.

To explore differences in the host response to Omicron and early circulating stains in patients with non-severe COVID-19, we conducted a literature search in PubMed using the search terms “COVID-19,” “plasma,” and “metabolite.” There were 19 relevant studies and we finally included four of them (Fig. 5A). Four studies were excluded because they were not age-matched to the COVID-19-infected group, six were excluded due to incomplete patient information or missing healthy controls, and five were excluded because they did not upload metabolite data. Finally, we included four studies that met our criteria: age-matched and complete metabolite data. Among the four included studies, two were from Taizhou and Guangzhou in China, and two were from the Seattle in USA and Novara in Italy. All patients in the four articles were infected with the original SARS-Cov-2 strain before July 2020. Differential metabolites (Fig. 5B, Table S7–S10) between patients with non-severe SARS-Cov-2 and healthy controls based on the criteria of *P* < 0.05, FC > 1.5 were screened from the differential metabolite data provided in four of the articles [9–12], and KEGG pathway enrichment analysis was performed (Fig. 5B, TableS11–S14). We then compared the enriched pathways and found none of the five studies shared any metabolic pathway (Fig. 5C). Different testing platforms, races, and regions may influence this result, and the number of samples collected (19 patients from Tianjin, 19 from Taizhou, 18 from Guangzhou, 84 from Novara, and 133 from Seattle, USA). Thirty-five of the 62 pathways were found only in patients with Omicron, indicating Omicron infection has unique metabolic characteristics. The ATP-binding cassette (ABC) transporter pathway, which is responsible for the ATP-powered translocation of many substrates across membranes, is one of the most important [43].

We compared the differential metabolites to understand better the metabolomic characteristics of Omicron infection (Fig. 5D, Table S7–S10). Patients infected with Omicron and those infected with SARS-Cov-2 at Taizhou, and Guangzhou, had more common differential metabolites. L-carnitine, propionylcarnitine, L-phenylalanine, L-5-hydroxytryptophan, *N*-acetylvaline, and *N*-acetylornithine levels were all upregulated in patients infected and patients from Guangzhou (Fig. 5D, Table S8). L-carnitine and propionylcarnitine are amino acids that can accelerate fatty acid oxidation. Therefore, L-carnitine can reduce the levels of inflammatory cytokines such as IL-6 and TNF-α [44]. In addition, Propionylcarnitine increased energy production by increasing the citric acid cycle [45]. L-phenylalanine, similar to tryptophan, regulates the T-cell immune response [46].

Interestingly, 4-hydroxybenzaldehyde levels were increased in the plasma of infected patients; however, they decreased in patients from Guangzhou. This may indicate that patients with Omicron experienced accelerated wound healing by activating focal adhesion signaling [47]. In contrast, the level of choline was downregulated in patients with Omicron due to macrophage activation. Compared to patients from Taizhou, only stearate was decreased, and the metabolite changes, including 1-methylhistidine, homoarginine, creatine, pipecolate, 5-hydroxyindoleacetate, and ornithine, were opposite in patients infected with Omicron. 1-methylhistidine is a histidine derivative that inhibits oxidative stress and attenuates inflammatory responses by downregulating TNF expression [18, 19]. Homoarginine, an arginine derivative, and ornithine participated in the urea cycle in the liver for detoxification. Higher homoarginine and ornithine levels indicated that the urea cycle was disrupted. Creatine has been shown to reduce inflammation [48] and reprogram macrophage polarization by suppressing IFN-γ, nevertheless promoting IL-4 expression [49]. Pipecolate has been previously shown to protect mammalian cells from oxidative stress [50]. These metabolite changes reflect the increased antioxidant capacity and anti-inflammatory effects of Omicron in patients compared to those of patients infected with the original SARS-CoV-2 strain. Patients from the USA and Italy had higher levels of glycocholate, 3-phosphoglycerate, triethanolamine, and glutamine than healthy controls, which was consistent with our findings. The increase in glycocholate may be related to liver cell damage and decreased glycocholate uptake in liver cells. 3-phosphoglycerate is a metabolic intermediate in glycolysis, and its increase correlates with glycolysis enhancement. Glutamine boosts the immune system by suppressing the inflammatory response [51]. In summary, while there are similar metabolite changes in patients infected with Omicron or the original SARS-CoV-2 strain, the metabolism in patients with Omicron has unique characteristics. Plasma metabolomics showed that Omicron infection mainly activates innate immunity and has a certain inhibitory effect on adaptive immunity. The body boosts antioxidants and energy supply to promote faster recovery.

## Discussion

In this study, we observed that, compared to healthy controls, mild and moderate COVID-19 patients presented with abnormal protein digestion and absorption, amino acid metabolism, and immune responses. There were some similarities; however, Omicron infections mainly activates innate immunity rather adaptive immunity compared with the original SARS-CoV-2 strain circulating from 2019.

Infectious disease development is closely linked to host metabolic processes [52, 53]. Viruses use metabolites to complete replication, and viral infection can disrupt metabolic processes [17, 54]. In contrast, metabolites can also regulate immune responses and influence viral infection [55, 56]. Therefore, maintaining metabolic homeostasis is the basis for the normal physiological function of the body, and metabolic homeostasis disturbances may promote virus infection. Research has shown that administration of the methionine derivative s-adenosylmethionine attenuates the cytokine storm caused by bacterial sepsis [57]. Consistent with our results, the S-adenosylmethionine level was elevated, and this level was associated with a favorable prognosis, possibly because their production reflects an attempt to attenuate the pathogenesis of COVID-19 [58]. Elevated methionine levels in Omicron indicate that the body uses less methionine, resulting in decreased T-helper cell proliferation and cytokine production. Moreover, plasma metabonomic analysis of COVID-19 patients also revealed evidence of significant activation of the kynurenine pathway [9]. Metabolites of kynurenate and kynurenine were enriched in COVID-19 patients. Other investigations indicated that kynurenic acid was negatively correlated with T-cell responses [20, 21]. In our study, the retinoic acid level was downregulated in the infected group compared to the control group. Retinoic acid, alone or in combination with stimuli that are ligands for the Toll-like receptor family, can enhance the adaptive immune response and promote antibody production by B cells [39, 40]. Our findings suggest that Omicron may primarily promote innate immunity while suppressing adaptive immunity. Unlike our findings, the threonine level was lower in patients with COVID-19 between March and June 2020, and its decrement could be related to catabolism [24]. Interestingly, the level of L-valine was lower in patients with COVID-19 than in healthy controls between March and April 2020, which could lead to vitamin B5 deficiency and impaired mitochondrial energy metabolism [12]. Infection from Omicron triggers inflammatory responses, and the body responds positively to the immune response through metabolic reprogramming to resist further transmission and destruction.

We further analyzed the unique changes in metabolism caused by the Omicron infection to previously published plasma metabolomics data from patients with COVID-19 infected with the original SARS-CoV-2 strain. We were based on comparing non-critically ill patients and healthy controls; however, the results of five studies showed that the number of differential metabolites and enriched pathways in the plasma was different in the patients. This may be due to the influence of factors such as different populations, sample sizes, blood collection time, and metabolomic platforms. Nevertheless, the plasma metabolomics of patients with Omicron shows that almost half of the metabolic pathways are unique. Specifically, we discovered that the three studies based on the Chinese population had more shared differential metabolites when we analyzed the shared differential metabolites between Omicron and the published data. These findings may be relevant to population and metabolomics platforms. These shared differential metabolites mainly involved amino acids and lipids. Similar to the original SARS-CoV-2 strain, the plasma metabolomics of patients with Omicron also revealed that the anti-inflammatory response (L-carnitine [44], L-5-hydroxyptophan [59], and glutamine [51]) were stimulated, and mitochondrial energy metabolism was enhanced (propionylcarnitine [45], *N*-acetylvaline [25], and 3-phosphoglycerate) to promote antiviral ability. However, the difference is that the macrophage regulation mechanism (creatine [49] and choline [36]) were different, neutrophil function (1-methylhistidine [18, 19]) and immune response caused by interferon (creatine [49] and L-5-hydroxyptophan [59]) were weak, antioxidant capacity (1-methylhistidine [18, 19] and pipecolate [50]) and liver detoxification function was enhanced (homoarginine and ornithine [22]). The few plasma metabolites shared by Omicron infections and the original strain reflected differences in metabolomics analysis, races, virus binding, host response, and population immunity during coronavirus mutation and transmission. The findings above show that when innate immunity is activated, the body actively reduces the inflammatory response and accelerates energy recovery. This may be one reason why patients with Omicron have milder clinical symptoms.

Omicron infection, like influenza, mainly affects the upper airways. Studies have shown that metabolomic analyses of patients with influenza reveal changes in amino acid and lipid metabolism. Patients with H1N1 had lower taurine concentrations than the control participants [60]. Taurine is an important compound involved in bile acid conjugation in the liver, suggesting its involvement in H1N1 infection. Unlike omicrons, decreased glycine, serine, and threonine concentration in patients with H1N1 may indicate viral consumption of these amino acids through viral metabolism [60]. In addition, low tryptophan levels were associated with death in hospitalized patients with influenza [61]. The downstream metabolites of tryptophan catabolism influence the inflammatory state. Kynurenic acid is a potent agonist of the orphan G-protein-coupled receptor GPR35, and its expression in T cells causes immunosuppression [62]. The interaction of GPR35 with kynurenic acid downregulates the pro-inflammatory effects of bacterial lipopolysaccharide in monocytes and macrophages [63–65]. In our study, high levels of kynurenic acid may indicate a suppressed immune response and weaker inflammation in the plasma of patients with Omicron. Moreover, the metabolomic analysis revealed that fatty acid metabolism might be suppressed during H7N9 infection. Therefore, palmitic acid levels in the blood may negatively correlate with the severity of lung inflammation following H7N9 infection [66]. Plasma metabolomics analysis of patients with Omicron revealed that phospholipid metabolites were downregulated during infection. Overall, the plasma metabolomic changes in patients with Omicron and influenza were similar, but they also had unique characteristics.

This study had some limitations. First, because the number of patients who underwent metabolomic analysis was small, the potential metabolites associated with disease progression found in this study are only suggestive and should be further investigated in large-scale studies. Second, the recovered patients had negative nucleic acid test results for 2 weeks; however, they were still post-acute, and the recovered group still showed abnormal protein digestion and absorption, amino acid metabolism, and immune responses compared to healthy control participants. Only L-valine, kynurenic acid, N-(2-hydroxyphenyl) acetamide, and choline levels returned to normal. The findings suggest that there are still some metabolic abnormalities in the body after 2 weeks of the negative nucleic acid test. It will take longer for the patient to return to homeostasis. Third, we selected patients with the original strain for comparative analysis; however, these metabolomics data were derived from different test platforms, and plasma sampling time and processing methods may differ, resulting in large differences in metabolites. Our findings revealed that patients infected with Omicron shared some common metabolic changes with patients infected with the original strain. The clinical symptoms of patients with Omicron were reduced; nevertheless, immune abnormalities caused by the Omicron virus persisted.

In conclusion, our findings suggest that omicron infection induces a weaker inflammatory and immune response, mainly innate immunity, than the original SARS-CoV-2 strain.

## Materials and methods

### Patients and samples

All participants were recruited from Haihe Hospital and First Central Hospital (Tianjin, China). We collected 83 plasma samples from 33 healthy participants, 19 infected patients, 31 recovered patients. All enrolled patients met the diagnostic criteria and clinical classification of the “Chinese Clinical Guidance for COVID-19 Pneumonia Diagnosis and Treatment (9th edition)”, published by the China National Health Commission. According to the clinical symptoms, confirmed patients with COVID-19 can be divided into the following four types: mild, moderate, severe, and critical. Mild type: mild clinical symptoms without signs of pneumonia on chest imaging; moderate type: respiratory symptoms and radiologic signs of pneumonia; severe type: any of the following four conditions: (1) shortness of breath, RR ≥ 30 times/min; (2) oxygen saturation ≤93% at rest; (3) partial arterial blood oxygen pressure (PaO2)/uptake oxygen concentration (FiO_2_) (PaO2/FiO2) ≤300 mmHg (1 mmHg = 0.133 kPa); (4) progressive exacerbation of clinical symptoms, and radiologic signs of significant progression of lesion >50% within 24–48 h; critical type: any of the following conditions: (1) respiratory failure with a necessity of mechanical ventilation; (2) shock; (3) other organ failures with a necessity of subjection to ICU monitoring and treatment. In our study, all the patients had mild and moderate COVID-19. Thirty-three age-matched healthy volunteers with no pulmonary abnormalities were recruited as controls, and they showed negative results in nucleic acid and antibody tests for SARS-CoV-2. Table 1 displays the clinical information of all participants. Infected patients were in the acute stage, while those who had recovered had negative nucleic acid test results for 2 weeks and were in the post-acute period. Venous blood (5 mL) was collected early in the morning, and the plasma was obtained by centrifugation at 4 °C (2000 rpm, 10 min). All plasma samples were stored in a freezer at −80 °C for metabolomic analysis.

### Metabolites extraction

The collected plasma samples were thawed and resuspended in pre-chilled 80% methanol by vortexing. Then the samples were incubated on ice for 5 min before being centrifuged at 15,000 × *g*, at 4 °C for 20 min. Some supernatants were diluted to a final concentration of 53% methanol using LC-MS grade water. The samples were then transferred to new Eppendorf tubes and centrifuged at 15,000 × *g*, at 4 °C for 20 min. Finally, the supernatant was then injected into the LC-MS/MS system for analysis.

### Ultra-performance liquid chromatography-tandem mass spectrometry analysis

UHPLC-MS/MS analyses were performed using a Vanquish UHPLC system (ThermoFisher, Germany) coupled with an Orbitrap Q Exactive^TM^ HF-X mass spectrometer (ThermoFisher, Germany) at Novogene Co., Ltd. (Beijing, China). The sample was injected into a Hypesil Gold column (C18) (100 mm × 2.1 mm, 1.9 μm) using a 17-min linear gradient at a flow rate of 0.2 mL/min. The eluents for the positive polarity mode were eluent A (0.1% FA in Water) and B (methanol). The eluents for the negative polarity mode were eluent A (5 mM ammonium acetate, pH 9.0) and B (methanol). The solvent gradient was set as follows: 2% B, 1.5 min; from 2% to 85% B, 3 min; from 85% to 100% B, 10 min; from 100%–2% B, 10.1 min; 2% B, 12 min. Exactive^TM^ HF-X mass spectrometer was set to positive/negative polarity with a spray voltage of 3.5 kV, a capillary temperature of 320 °C, a sheath gas flow rate of 35 psi, an auxiliary gas flow rate of 10 L/min, an S-lens RF level of 60, and Aux gas heater temperature of 350 °C.

### Metabolite identification, and quantitation

The raw data files generated by UHPLC-MS/MS were processed using Compound Discoverer 3.1 (CD3.1, ThermoFisher) to perform peak alignment, peak picking, and quantitation for each metabolite. The main parameter was set as follows: retention time tolerance of 0.2 min; actual mass tolerance of 5 ppm; signal intensity tolerance of 30%; signal/noise ratio of 3; and minimum intensity. Peak intensities were normalized concerning total spectral intensity. The normalized data were used to predict the molecular formula based on additive ions, molecular ion peaks, and fragment ions. The peaks were then matched using the mzCloud (https://www.mzcloud.org/), mzVault, and MassList databases to obtain accurate qualitative and quantitative results.

### Quality control of metabolomics analysis

As described previously [15, 67], we mixed an equal volume of each plasma sample as a separate sample for quality control (QC) of metabolomics analysis, to balance the chromatography-mass spectrometry system and monitor instrument status, and evaluate system stability throughout the experiment. The blank sample was also set to remove background ions. The QC sample was split into aliquots which were then tested on the machine before, during, and after the LC-MS/MS loading of the experimental samples. We obtained 11 metabolomic profiling from the QC sample aliquots. Pearson correlation coefficients between QC sample aliquots were calculated based on the relative quantification of the metabolites. We calculated the coefficient of variance (CV) values of the metabolites in the QC sample, and metabolites whose CV values were larger than 0.3 were excluded. Before statistical data analysis, a sample normalization was performed as previously described [67], original quantification of metabolites/(sum of metabolite quantifications/sum of metabolite quantifications in QC1).

### Differential metabolites and pathway analysis

The KEGG database (https://www.genome.jp/kegg/pathway.html), LIPIDMaps database (http://www.lipidmaps.org/), and HMDB database (https://hmdb.ca/metabolites) were used to annotate the metabolites. Multivariate and principal component analyses (PCA) were performed to obtain the VIP values for each metabolite. Univariate analysis was performed using a t-test to calculate the statistical significance and fold-change (FC) for each metabolite between the two groups. Metabolites with VIP > 1, False Discovery Rate (FDR) < 0.05, and FC > 1.5 were considered differential metabolites. Volcano plots were screened based on log_2_(FC) and log_10_(FDR) of metabolites using ggplot2 in the R language. Differential metabolite clustering heat maps were normalized using z-scores of the intensity zone and plotted with the Pheatmap package in the R language. Using the Kyoto Encyclopedia of Genes and Genomes (KEGG, http://www.genome.jp/kegg/) database, we performed KEGG pathway enrichment analysis based on differential metabolites to explore the biological processes of the disease. The metabolic pathway was considered statistically significant when the *p* value was <0.05. Cluster analysis of metabolite intensity from different patients was performed using Mfuzz v.2.46.0, which can identify underlying time-series patterns of expression profiles and cluster metabolites with similar patterns to clarify the dynamic patterns of metabolites and their functional linkages.

### Statistical analysis

The normality of the data distributions was assessed using the Kolmogorov–Smirnov test. Normally distributed data are presented as mean (± standard deviation), whereas abnormally distributed data are presented as median (± interquartile range), and categorical variables are presented as frequencies (%). Statistical significance was calculated for metabolites in three groups (healthy, infected, and recovered) using the Kruskall–Wallis and Dunn post-hoc tests when data were not normally distributed. An ANOVA with a post hoc test was used when data were normally distributed, and a *p* value < 0.05 was considered statistically significant.

## Supplementary information

Legends to supplementary figures

Figure S1

Supplementary tables

aj-checklist

## Data Availability

The data that support the findings of this study are available from the corresponding author upon reasonable request. The metabolomics data have been deposited to the ProteomeXchange Consortium (http://proteomecentral.proteomexchange.org/cgi/GetDataset?ID=PXD040370) with the dataset identifier IPX0005977000.
