# Supplementary material for: Plasma metabolomic characterization of SARS-CoV-2 Omicron infection
Source: Cell Death Dis. 2023 Apr 19;14(4):276. doi: 10.1038/s41419-023-05791-3 (PMC10113737; doi:10.1038/s41419-023-05791-3)
Supplement: Supplementary file 1 — Legends to supplementary figures [file 41419_2023_5791_MOESM1_ESM.docx]

**Legends to supplementary figures**

**Fig S1. Expression profiles were analyzed according to metabolic abundance between patients with COVID-19 and healthy controls.**

A. The result of cluster analysis in processing conditions (Healthy-Infected-Recovered) by the Mfuzz package in M4–M6. The color bar represents the Z score change from −1 to 1.

B. Barplot for functional KEGG enrichment result for metabolites. *P-value* < 0.05 was identified as a significantly changed pathway. The X-axis shows the *p-value* of each term, and the Y-axis shows the function terms.

C．Heatmap visualization of metabolites in M4–M6 under the processing conditions (healthy, infected, recovered). The color bar represents the relative intensity of identified metabolites from −4 to 4.
