# Supplementary figures and images for: Plasma metabolomic characterization of SARS-CoV-2 Omicron infection
Source: Cell Death Dis. 2023 Apr 19;14(4):276. doi: 10.1038/s41419-023-05791-3 (PMC10113737; doi:10.1038/s41419-023-05791-3)

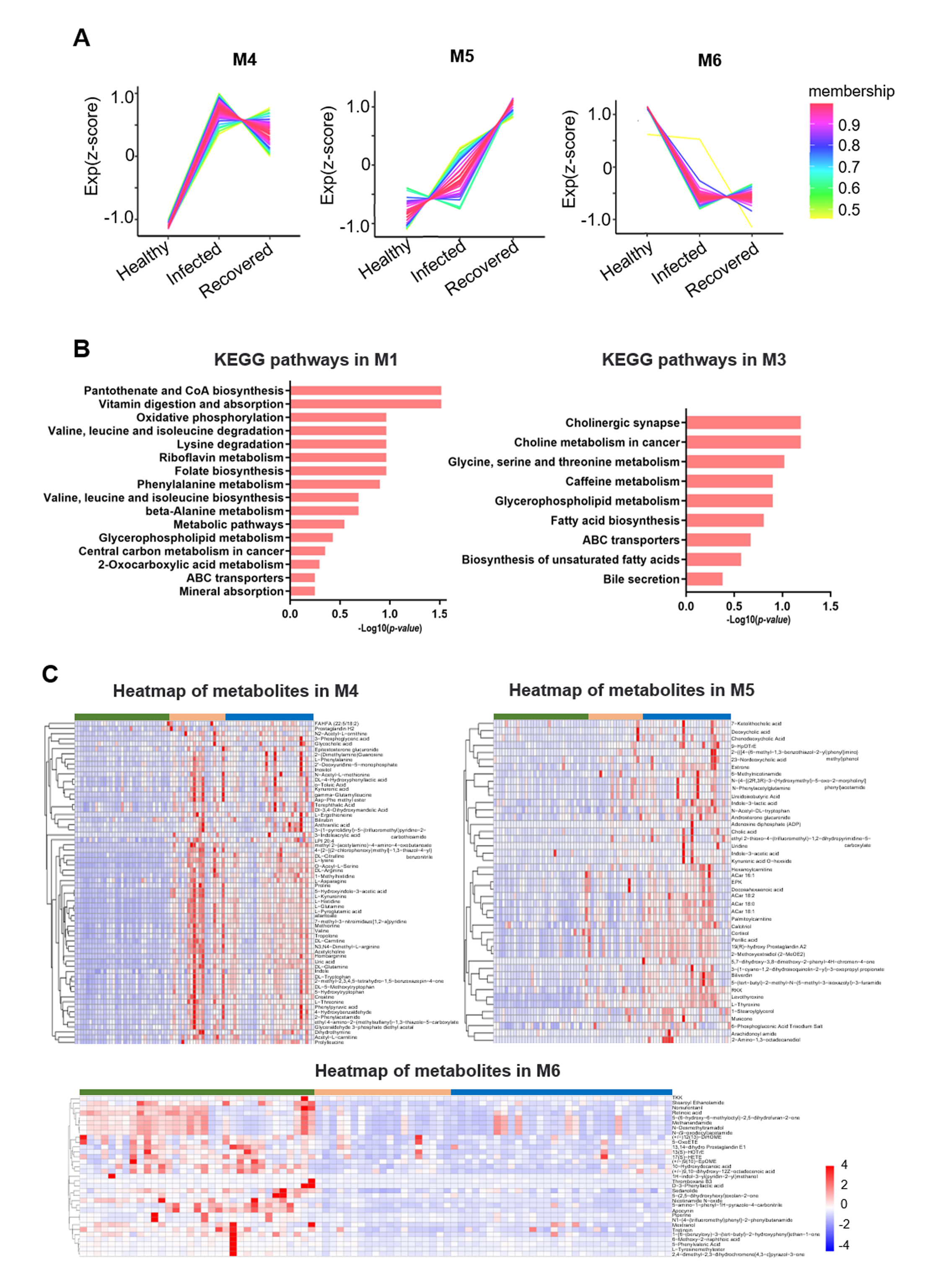

Supplement: Supplementary file 2 — Figure S1 [file 41419_2023_5791_MOESM2_ESM.tif]
